# Single‐Molecule Force Spectroscopy Reveals Stability of mitoNEET and its [2Fe2Se] Cluster in Weakly Acidic and Basic Solutions

**DOI:** 10.1002/open.202200056

**Published:** 2022-05-24

**Authors:** Jing‐Yuan Nie, Guo‐Bin Song, Yi‐Bing Deng, Peng Zheng

**Affiliations:** ^1^ State Key Laboratory of Coordination Chemistry Chemistry and Biomedicine Innovation Center (ChemBIC) School of Chemistry and Chemical Engineering Nanjing University Nanjing Jiangsu 210023 P. R. China

**Keywords:** AFM, iron-sulfur protein, mitoNEET, single-molecule force spectroscopy

## Abstract

The outer mitochondrial membrane protein mitoNEET (mNT) is a recently identified iron‐sulfur protein containing a unique Fe_2_S_2_(His)_1_(Cys)_3_ metal cluster with a single Fe−N(His87) coordinating bond. This labile Fe−N bond led to multiple unfolding/rupture pathways of mNT and its cluster by atomic force microscopy‐based single‐molecule force spectroscopy (AFM‐SMFS), one of most common tools for characterizing the molecular mechanics. Although previous ensemble studies showed that this labile Fe−N(His) bond is essential for protein function, they also indicated that the protein and its [2Fe2S] cluster are stable under acidic conditions. Thus, we applied AFM‐SMFS to measure the stability of mNT and its cluster at pH values of 6, 7, and 8. Indeed, all previous multiple unfolding pathways of mNT were still observed. Moreover, single‐molecule measurements revealed that the stabilities of the protein and the [2Fe2S] cluster are consistent at these pH values with only ≈20 pN force differences. Thus, we found that the behavior of the protein is consistent in both weakly acidic and basic solutions despite a labile Fe−N bond.

## Graphical abstract

**Applying AFM‐Single‐Molecule Force Spectroscopy (SMFS)** to MitoNEET, an iron‐sulfur protein containing a unique Fe_2_S_2_(His)_1_(Cys)_3_ metal cluster with a single Fe−N(His87) bond, was used to measure its stability. Both MitoNEET and its constituent [2Fe2Se] are stable cluster at pH 6, 7, and 8. These result show that the behavior of the protein is consistent in both weakly acidic and basic solutions despite its labile Fe−N bond.

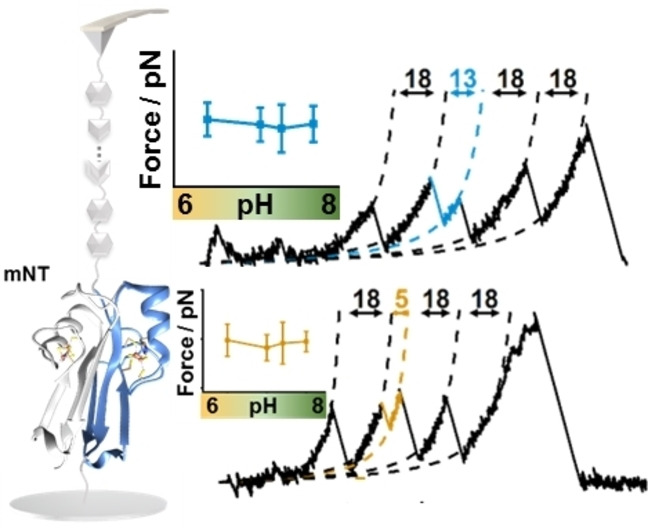

## Introduction

Iron‐sulfur proteins occupy a crucial position in a wide range of biological processes, including nitrogen fixation, photosynthesis, and respiration.^[1]^ Recently, a mitochondrial protein mitoNEET (mNT), as the first member of a novel NEET protein family, has been identified to harbor a unique (Cys)_3_(His)_1_ [2Fe2S] cluster.^[2]^ The protein is believed to be involved in the homeostasis of mitochondrial iron,^[3]^ in which the single Fe−N bond plays an important role. Indeed, our previous atomic force microscopy‐based single‐molecule force spectroscopy (AFM‐SMFS) work indicated that the Fe^III^−N(His87) bond in the cluster is labile, which resulted in multiple rupture pathways of the metal cluster in mNT under physiological conditions (pH 7.4).^[4]^ Nevertheless, previous ensemble studies showed the [2Fe2S] cluster to be stable in a weakly acidic solution (>pH 5),[^2b^, ^5^] regardless of the weak Fe−N bond. Considering the Fe−N bond is pH‐sensitive, we utilize AFM‐SMFS to explore whether the stability of the protein and the whole metal cluster are consistent under weakly acidic and basic solutions (pH 6, 7 and 8).

**Figure 1:**
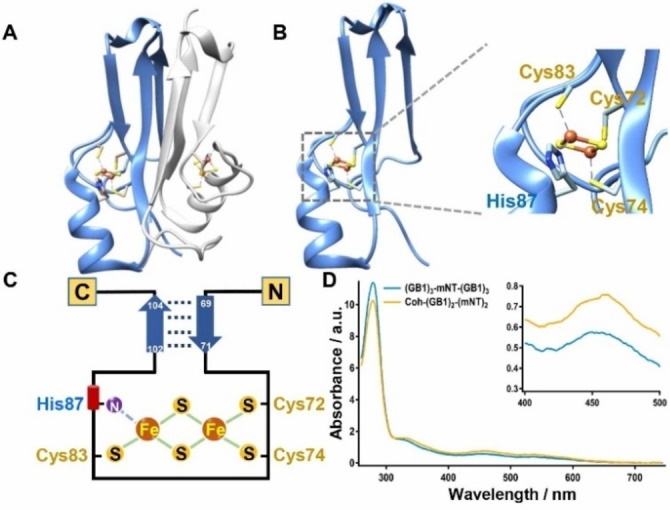
Schematic depiction of mNT. (A) The structure of the mNT homodimer. (B) Predicted structure of the mNT monomer containing a [2Fe2S] cluster, which is coordinated with Cys72, Cys74, Cys83, and His87. (C) Schematic depicition of mNT monomer showing two β‐strands and a metal cluster. (D) The UV‐Vis spectrum of Cohesion‐(GB1)_2_‐(mNT)_2_ (colored in khaki) and (GB1)_3_‐mNT‐(GB1)_3_ (colored in blue) showing the characteristic absorption at 458 nm due to the [2Fe2S] cluster.

MitoNEET, existing as a dimer, is an integral outer mitochondrial membrane protein with a soluble cytosolic domain from residues 33 to 108 (Figure 1A).^[6]^ Each monomer is comprised of two domains: a cluster‐binding domain (residues 72–87) and a β‐cap domain (residues 56–61, 68–71, and 98–104) (Figure 1B). The [2Fe2S] cluster in mNT is uniquely coordinated by three cysteines (Cys72, Cys74, and Cys83) and one histidine (His87) (Figures 1B–C). Thus, the protein solution appears reddish and shows a characteristic UV‐Vis absorption peak at 458 nm (Figure 1D).

Single‐molecule force spectroscopy techniques, such as single‐molecule AFM, optical tweezers, and magnetic tweezers, can mechanically manipulate an individual molecule, leading to the rupture of intra‐ or intermolecular bond/interaction and the emergence of an intermediate state.^[7]^ Because of its high force range and excellent length resolution, AFM has been widely used in the study and measurement of protein (un)folding, ligand‐receptor interactions, and chemical bonds in an aqueous solution.^[8]^ Thus, we have used and continue to employ AFM‐SMFS to study the stability of the metal cluster in mNT in different environments.^[9]^

## Results and Discussion

**Figure 2:**
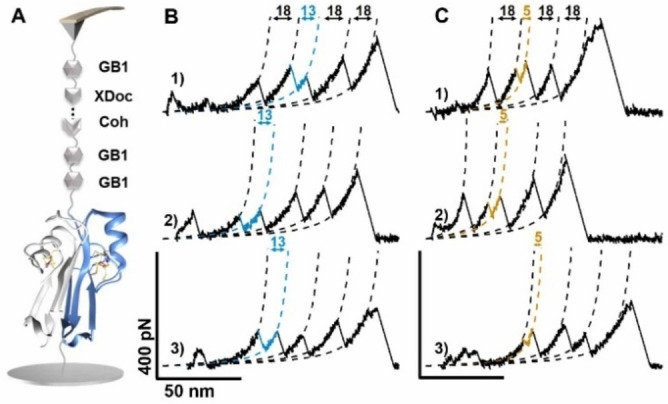
Single‐molecule force spectroscopy experiments for Coh‐(GB1)_2_‐(mNT)_2_ at pH 6–8; Coh is the abbreviation for protein cohesion. (A). Setup of AFM experiment showing that Coh‐(GB1)_2_‐(mNT)_2_ is picked up by the GB1‐XDoc‐coated AFM tip through [Coh‐XDoc] protein‐protein interaction. (B–C) Typical force‐extension curves of the protein showing peaks with ΔL_c_ of 13 nm (colored in blue) and 5 nm (colored in khaki), which are attributed to the one‐step unfolding of mNT and rupture of the [2Fe2S] cluster, respectively. Curve 1 was obtained at pH 6, curve 2 at pH 7, and curve 3 at pH 8.

To explore the stability of mNT under a broader range of pH values, an mNT dimer construct, Coh‐(GB1)_2_‐(mNT)_2_‐NGL, whose unfolding mechanism is well‐explored at pH 7.4, was used for this study (Figure 2A). Protein ligase *Oa*AEP1 (*Oldenlandia affinis* asparaginyl endopeptidase 1) was used to site‐specifically immobilize the protein in the AFM measurement system.^[10]^ The ligase connects two proteins/peptides between its N‐terminal dipeptide GL (Gly‐Leu) and C‐terminal tripeptide NGL (Asp‐Gly‐Leu). Therefore, Coh‐(GB1)_2_‐(mNT)_2_‐NGL was immobilized on a GL‐coated substrate. A GB1‐XDoc functionalized AFM tip was used (XDoc is the abbreviation for protein complex XModule‐dockerin). Thus, when the tip approached the substrates, a specific [Coh : XDoc] protein‐protein interaction led to a close force loop between the tip and coverslip as tip‐GB1‐[XDoc : Coh]‐(GB1)_2_‐(mNT)_2_‐substrate (Figure 2A). Here, GB1 shows a contour length increment (ΔL_c_) of 18 nm upon unfolding. The rupture of [XDoc : Coh] finally shows a high force peak (≈500 pN). These findings can be used characteristic signals for identifying the single‐molecule event.

**Figure 3:**
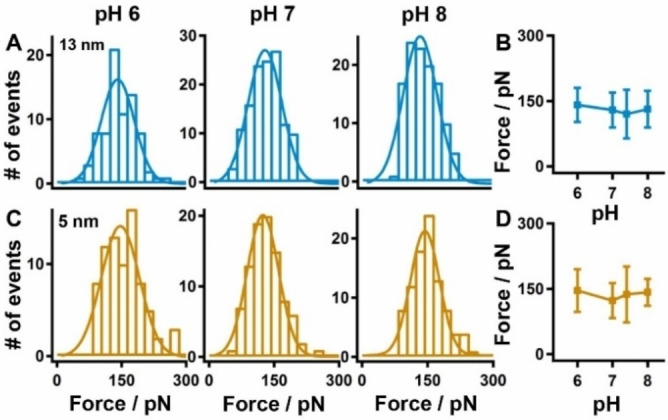
Statistics of unfolding force for mNT in its functional dimer form from pH 6 to 8. The rupture force for the 13 nm peak (A–B) and the 5 nm peak (C–D) at different pH showed a similar force value around 130 pN.

Stretching the polyprotein by single‐molecule AFM under three pH conditions (pH 6, 7, and 8) resulted in typical force‐extension curves, containing three peaks from GB1 and additional peak(s) from mNT as expected (Figures 2B–C). First, one‐step unfolding events of mNT with a ΔL_c_ of 13 nm were observed (Figure 2B, colored in blue, curve 1 at pH 6, curve 2 at pH 7, and curve 3 at pH 8), fitting by the worm‐like chain (WLC) model of elastic polymers. Theoretically, the ΔL_c_ of the complete unfolding of mNT equals to the extension of 37 aa between residues 68 to 104 (37 aa×0.36‐0.46=12.9 nm; 0.46 nm is the distance between residues 68 and 104 in the folded mNT), which agrees with the experimental value (12.5±1.0 nm, at pH 6). The unfolding force was 140±54 pN (avg. ± stdv., *n*=78) at pH 6, 128±55 pN (*n*=128) at pH 7, and 133±55 pN (*n*=117) at pH 8 (Figures 3A–B and Table 1), which is comparable to previous result (120±56 pN) at pH 7.4. Thus, the stability of mNT is quite similar from pH 6 to 8 (Figure 3B, Table 1).

**Table 1:** The unfolding force [pN] of mNT at different pH values.

pH	Dimer		Monomer	
	13 nm (*n*)^[a]^	5 nm (*n*)	13 nm (*n*)	5 nm (*n*)
6	140±54 (*78*)	147±62 (*77*)	125±80 (*120*)	113±70 (*104*)
7	128±55 (*128*)	124±51 (*93*)	134±82 (*134*)	107±41 (*78*)
7.4^[b]^	120±56	137±64	105±93	82±51
8	133±55 (*117*)	143±46 (*93*)	144±73 (*168*)	106±67 (*102*)

[a] *N* represents the total number of data points and the error values represent the dispersion degree of these data points. [b] The unfolding forces at pH 7.4 was obtained in our previous work.^[4]^

In addition, the stepwise unfolding of mNT was also observed, showing different unfolding pathways with peaks of ΔL_c_ shorter than 13 nm (Figures 2C and S2, Supporting Information). This provides detailed information about the stability of the [2Fe2S] cluster. Here, we focused on the rupture events of the [2Fe2S] cluster with ΔL_c_ of 5 nm (Figure 2C, colored in khaki, curve 1 at pH 6, curve 2 at pH 7, and curve 3 at pH 8). This peak orginated from the one‐step rupture of the intact metal cluster, with ΔL_c_ corresponding to the extension of residues 72–87 (16 aa×0.36–0.5=5.26 nm). The unfolding force of 5 nm peak amounted to 147±62 pN (avg. ±stdv., *n*=77) at pH 6, 124±51 pN (*n*=93) at pH 7, and 143±46 pN (*n*=93) at pH 8 (Figures 3C–D, Table 1), which is comparable to the previous result at pH 7.4 Thus, similar to the unfolding force of mNT at pH 6 to 8, the rupture force of the cluster remains similar in this pH range. (Figure 3D, Table 1). Furthermore, other stepwise unfolding/rupture pathways of mNT/the cluster were observed, agreeing with previous observations at pH 7.4 (Figure S2A).

**Figure 4:**
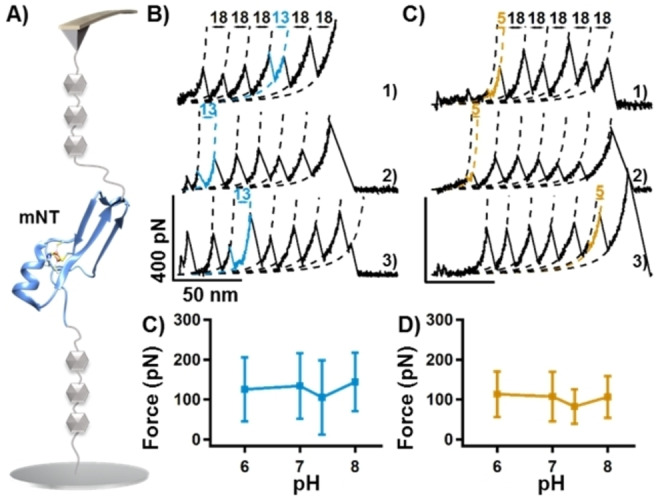
Single‐molecule force spectroscopy experiments for (GB1)_3_‐mNT‐(GB1)_3_ in a buffer with pH 6–8. (A) Setup of an AFM experiment showing that mNT monomer was stretched using a (GB1)_3_‐mNT‐(GB1)_3_ construction. (B–C) Typical force‐extension curves with ΔL_c_ of 13 nm (colored in blue) and 5 nm (colored in khaki), which was attributed to the one‐step unfolding of mNT and the rupture events of the [2Fe2S] cluster, respectively. Curve 1 was obtained at pH 6, curve 2 at pH 7, and curve 3 at pH 8. Statistical analysis of force versus pH (C–D) furthermore showed a stable [2Fe2S] cluster from pH 6 to 8.

To further confirm the stability of the [2Fe2S] cluster in mNT from pH 6 to 8, we also studied the mNT monomer using a (GB1)_3_‐mNT‐(GB1)_3_ construct (Figure 4A). Here, three GB1s were flanked on each side of the mNT monomer to prevent dimer formation. As expected, previously observed peaks in the dimer unfolding were all observed (Figure 4B–C, 13 nm colored in blue, 5 nm colored in khaki; curve 1 at pH 6, curve 2 at pH 7, and curve 3 at pH 8). Again, the unfolding force for these peaks was similar from pH 6 to 8 (Figures 4C–D and Table 1), indicating that the intact metal cluster of mNT shows consistent stability. Whether as a monomer or a dimer, the metal cluster of mNT thus showed certain stability under weak acidic and basic conditions by AFM‐SMFS.

Here, protein domain GB1 is used in the polyprotein as a single molecule marker which does not affect the result of mNT. First, the unfolding event for each protein (mNT and GB1) is independent, as shown by the sequential unfolding force peak in the force‐extension curve. If there is any protein‐protein interaction between them, this weak intermolecular interaction (<50 pN) will be broken before the protein is unfolded (>100 pN). Moreover, the solution pH does not affect GB1 either. Previous AFM measurements on GB1 from pH 4 to 8.5 showed that the unfolding force of GB1 is consistent, with a force of ≈180 pN.^[9a]^ Indeed, this polyprotein strategy incorporating GB1 as a single‐ molecule marker has been widely used to study many other metalloproteins, such as the iron‐sulfur proteins rubredoxin, ferredoxin, and metallothionein.[^8a^, ^8f^, ^9b^] Previously, we performed the AFM experiment on mNT under physiological conditions at pH 7.4 and found the Fe−N bond is labile, leading to multiple rupture pathways of the metal cluster and protein.

It is noted that the Fe−N bond is pH sensitive. Protons (H^+^) will compete for the nitrogen atom (N) in an acidic solution, while hydroxyl (OH^−^) ions will compete for the ferric ion. Thus, both acidic and basic solutions might destabilize the cluster and thus disable the function of this protein. However, ensemble studies showed that mNT retains its function in mitochondria where the pH is not always neutral. To further verify the function of the Fe−N bond in mNT and the overall stability of the protein, we performed this experiment and found that the stability of the cluster and protein is consistent under acidic and basic conditions. This result indicates that the protein structure protects the cluster and the labile Fe−N bond, and the protein and metal cluster collectively fulfill the protein function, which has not been demonstrated before.

## Conclusion

In this work, we measured the stability of mNT and its [2Fe2S] cluster under pH 6, 7, and 8 at the single‐molecule level by AFM‐SMFS. Although the multiple unfolding pathways and intermediates were still observed due to the labile Fe−N bond, the stability of mNT and its [2Fe2S] cluster measured are consistent in this pH range, with only ≈20 pN force difference. In addition, single‐molecule measurements on both the functional mNT dimer and a mNT monomer confirmed that the protein and its central cluster are stable at pH 6, agreeing with the previous conclusion from ensemble studies.

## Experimental Section

### Protein Engineering

The fusion protein Coh‐(GB1)_2_‐mNT, GST‐mNT, and (GB1)_3_‐mNT‐(GB1)_3_ were constructed using standard molecular biology techniques and overexpressed in the *E. coli* BL21(DE3) strain, as previously reported. Cultures from a single colony were grown in the M9 medium and induced at 23 °C for 18 h after adding 1 mm isopropyl β‐D‐thiogalactoside (IPTG) and 1 mm FeCl_3_. Then (GB1)_3_‐mNT‐(GB1)_3_ and Coh‐(GB1)_2_‐mNT were purified by Co^2+^‐affinity chromatography. GST‐mNT was purified by GST‐affinity chromatography.

### Single‐Molecule AFM Experiments

Single‐molecule AFM experiments were carried out on the commercial NanoWizard4 AFM (JPK Germany). The AFM cantilever was calibrated in Tris buffer (50 mm Tris, 150 mm NaCl) by the equipartition theorem. All experiments were performed under a constant pulling speed of 400 nm/s.

For the mNT dimer experiment using Coh‐(GB1)_2_‐mNT construct, a site‐specific protein immobilization method and a cohesion (Coh)‐dockerin (Doc) protein‐protein interaction pair as molecular handle were utilized in the AFM system to ensure that protein of interest was fully unfolded during single‐molecule experiments.[^10a^, ^11^] During the experiments, AFM tips were pressed on the substrate with a contact force of 500 pN and precisely stretched the mNT through [Coh:Doc] interaction. After recording each force‐extension curve, the tips moved horizontally by 50 nm for the subsequent measurement. Only curves containing three unfolding events of fingerprint GB1 and a high rupture force from the break of [Coh:Doc] interaction were selected for analysis by the worm‐like chain (WLC) model.

For the mNT monomer experiment using the (GB1)_3_‐mNT‐(GB1)_3_ construct, a classic non‐specific protein absorption method was used. AFM tips approached the protein‐deposited cover glasses with a 500–600 pN contact force and randomly picked up a polyprotein by non‐specific interaction. During the tips departed from the glasses, the polyprotein unfolded, leading to the rupture of the iron‐sulfur cluster. Finally, the polyprotein detached and the tips moved to the next location for repeated cycles. GB1, a single‐molecular fingerprint with exact unfolding force and ΔL_c_, flanked both sides of mNT. Therefore, a force‐extension curve with more than four GB1 unfolding events was selected and analyzed. The force‐extension curves were fitted by the WLC model [Eq. 1].

(1) $$\;F\left(x\right)=\;\frac{k_{B}T}{p}\left[\frac{1}{4}{\left(1-\frac{x}{L_{c}}\right)}^{-2}-\frac{1}{4}+\frac{x}{L_{c}}\right]$$

*p* denotes the persistence length. L_c_ is the contour length of the polymer/protein. *T* is the absolute temperature in K. *k_B_* is the Boltzmann constant.

## Conflict of interest

The authors declare no conflict of interest.

1

## Supporting information

As a service to our authors and readers, this journal provides supporting information supplied by the authors. Such materials are peer reviewed and may be re‐organized for online delivery, but are not copy‐edited or typeset. Technical support issues arising from supporting information (other than missing files) should be addressed to the authors.

Supporting Information

## Data Availability

The data that support the findings of this study are available from the corresponding author upon reasonable request.
