# Supplementary material for: Single‐Molecule Force Spectroscopy Reveals Stability of mitoNEET and its [2Fe2Se] Cluster in Weakly Acidic and Basic Solutions
Source: ChemistryOpen. 2022 May 24;11(5):e202200056. doi: 10.1002/open.202200056 (PMC9127745; doi:10.1002/open.202200056)
Supplement: Supplementary file 1 — Supporting Information [file OPEN-11-e202200056-s001.pdf]

ChemistryOpen

Supporting Information

Single-Molecule Force Spectroscopy Reveals Stability of mitoNEET and its [2Fe2Se] Cluster in Weakly Acidic and Basic Solutions

Jing-Yuan Nie, Guo-Bin Song, Yi-Bing Deng, and Peng Zheng*

This Supplementary Information Includes:

Supplementary Methods **2**

Protein engineering 2

Protein immobilization 2

Supplementary Note **4**

Protein sequences 4

Supplementary Figures **6**

Figure S1. 6

Figure S2. 6

Figure S3. 7

Supplementary Methods

Protein engineering

The fusion protein $(GB1)_3$-mNT-$(GB1)_3$, Coh-$(GB1)_2$-mNT and GST-mNT were constructed using standard molecular biology techniques and overexpressed in the *E. coli* BL21(DE3) strain, as previously reported. $(GB1)_3$-mNT-$(GB1)_3$ and Coh-$(GB1)_2$-mNT were purified by Co^{2+}-affinity chromatography. After washing (50 mM Tris, 200 mM NaCl, 2 mM imidazole, pH 7.4), the chimeric protein was eluted in the buffer (50 mM Tris, 200 mM NaCl, 500 mM imidazole, pH 7.4). GST-mNT was purified by GST-affinity chromatography. Washing by washing buffer (50 mM Tris, 200 mM NaCl, pH 7.4), GST-mNT was eluted by elution buffer (50 mM Tris, 200 mM NaCl, 20 mM glutathione (GSH), pH 7.4). All fusion proteins appeared red with a characteristic UV-Vis absorption peak at 458 nm in the presence of the 2Fe2S cluster.

Besides, the mNT dimer was cultivated as reported. Coh-$(GB1)_2$-mNT and GST-mNT were incubated at a ratio of 1:4 for 48 h. Then, the target dimer-protein Coh-$(GB1)_2$-$(mNT)_2$-GST was purified with GST column and cleaved by 3C protease to remove the GST tag. Finally, protein dimer was further purified with a gel filtration column.

Protein immobilization

For $(GB1)_3$-mNT-$(GB1)_3$, the coverslip was cleaned by plasma treatment. And ~ 10 μL protein sample with a concentration of ~ 0.6 mg/mL, was deposited on the cleaning coverslip and incubated at 25°C for 0.5 h. After that, 1.5 mL Tris buffer was added to perform AFM experiments. Si_3N_4 cantilever (MLCT-BIO-DC, Bruker) was directly used for AFM experiments with only plasma treatment.

For Coh-$(GB1)_2$-$(mNT)_2$, AFM tips and coverslip were coated by amine groups with 3-aminopropyltriethoxysilane (APTES) after plasma treatment. Then the tips and cover glasses were coated with azide group by azidation reacting solution (4 mM $ImSO_2N_3$, 8 mM K_2CO_3, 40 μM $CuSO_4$). Then, the maleimide groups were introduced on the surface of tips and cover glasses by adding DBCO-$(PEG)_4$-maleimide. Cys-ELP_{20}-NGL/GL-ELP_{20}-Cys were reacted on the tips/cover glasses.

Finally, Coh-$(GB1)_2$-$(mNT)_2$ containing a C-terminal NGL were covalently immobilized on the cover glasses by ligase *Oa*AEP1(Cys274Ala). The GB1-XDoc were linked to the NGL-coated AFM tips with an N-terminal GL.

Supplementary Note

Protein sequences

$(His)_6$-Coh-$(GB1)_2$-**mNT**

MRGSHHHHHHGSMGTALTDRGMTYDLDPKDGSSAATKPVLEVTKKVFDTA
ADAAGQTVTVEFKVSGAEGKYATTGYHIYWDERLEVVATKTGAYAKKGAA
LEDSSLAKAENNGNGVFVASGADDDFGADGVMWTVELKVPADAKAGDVYP
IDVAYQWDPSKGDLFTDNKDSAQGKLMQAYFFTQGIKSSSNPSTDEYLVKAN
ATYADGYIAIKAGEPRSMDTYKLILNGKTLKGETTTEAVDAATAEKVFKQYA
NDNGVDGEWTYDDATKTFTVTERSMDTYKLILNGKTLKGETTTEAVDAATA
EKVFKQYANDNGVDGEWTYDDATKTFTVTERS**RFYVKDHRNKAMINLHIQ**
KDNPKIVHAFDMEDLGDKAVYCRCWRSKKFPFCDGAHTKHNEETGDNV
GPLIIKKKETRSVPGVGVPGVGVPGEGVPGVGVPGVGVPGVGVPGVGVPGE
GVPGGLRSNGL

GST-**mNT**

MSPILGYWKIKGLVQPTRLLLEYLEEKYEEHLYERDEGDKWRNKKFELGLEF
PNLPYYIDGDVKLTQSMAIIRYIADKHNMLGGCPKERAEISMLEGAVLDIRYG
VSRIAYSKDFETLKVDFLSKLPEMLKMFEDRLCHKTYLNGDHVTHPDFMLYD
ALDVVLYMDPMCLDAFPKLVCFKKRIEAIPQIDKYLKSSKYIAWPLQGWQAT
FGGGDHPPKGS**RFYVKDHRNKAMINLHIQKDNPKIVHAFDMEDLGDKAV**
YCRCWRSKKFPFCDGAHTKHNEETGDNVGPLIIKKKETRS

$(GB1)_3$-**mNT**-$(GB1)_3$

MRGSHHHHHHGSMDTYKLILNGKTLKGETTTEAVDAATAEKVFKQYANDN
GVDGEWTYDDATKTFTVTERSMDTYKLILNGKTLKGETTTEAVDAATAEKV
FKQYANDNGVDGEWTYDDATKTFTVTERSMDTYKLILNGKTLKGETTTEAV

DAATAEKVFKQYANDNGVDGEWTYDDATKTFTVTERS**RFYVKDHRNKAM INLHIQKDNPKIVHAFDMEDLGDKAVYCRCWRSKKFPFCDGAHTKHNEE T**GDNVGPLIIKKKETRSMDTYKLILNGKTLKGETTTEAVDAATAEKVFKQYA NDNGVDGEWTYDDATKTFTVTERSMDTYKLILNGKTLKGETTTEAVDAATA EKVFKQYANDNGVDGEWTYDDATKTFTVTERSMDTYKLILNGKTLKGETTT EAVDAATAEKVFKQYANDNGVDGEWTYDDATKTFTVTERS

Supplementary Figures

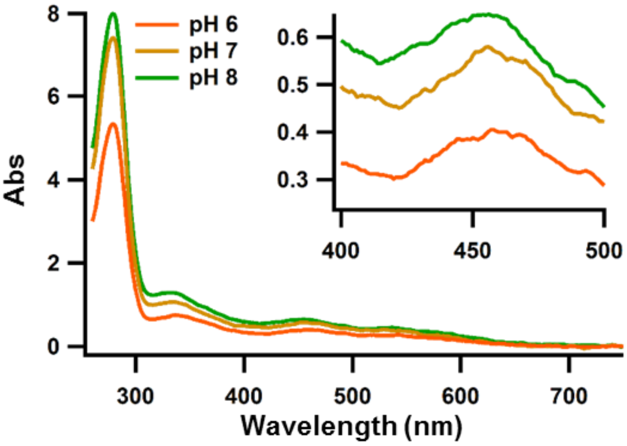

Figure S1. The UV-Vis spectra of Coh-(GB1)$_2$-(mNT)$_2$ showed the characteristic absorption at 458 nm (insert) from the [2Fe-2S] cluster of mNT in the buffer with different pH (pH 6 colored in orange, pH 7 colored in khaki, pH 8 colored in green).

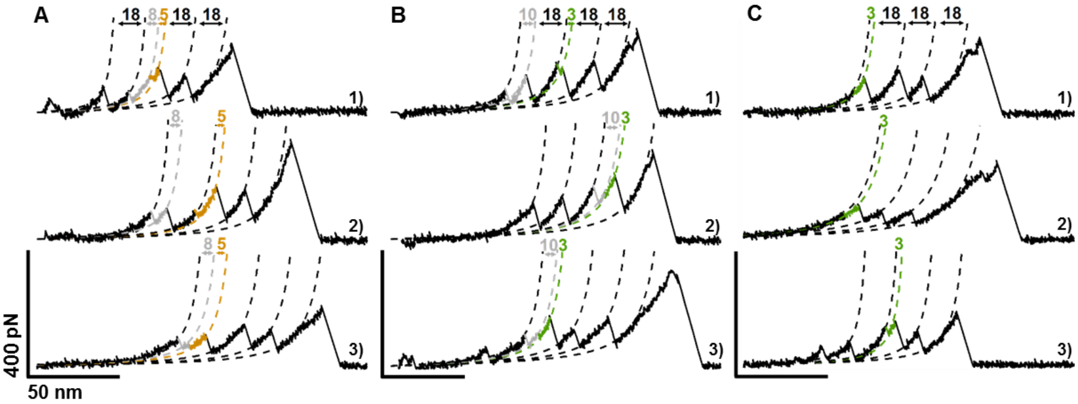

Figure S2. More stepwise unfolding curves of site-specific AFM experiments showed different unfolding pathways for holo mNT. Curve 1 was obtained in pH 6, curve 2 in pH 7, and curve 3 in pH 8.

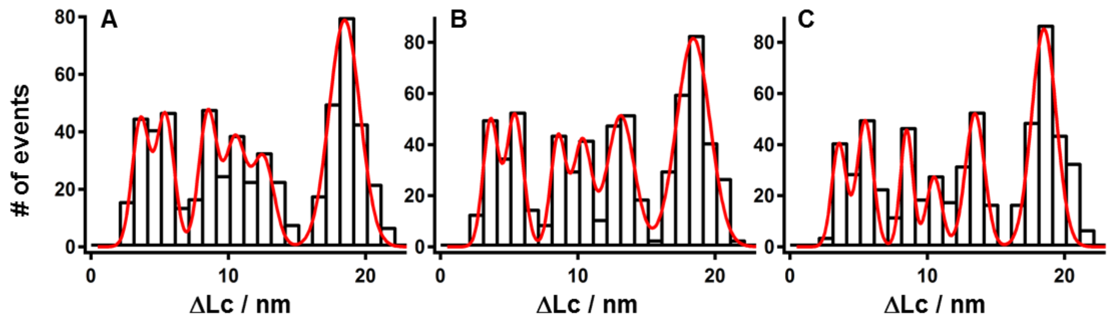

Figure S3. ΔLc histograms of Coh-$(GB1)_2$-$(mNT)_2$ under different pH. (A) pH 6; (B) pH 7; (C) pH 8.
